# Depressive symptoms, insomnia, and burnout among health-care workers in an integrated medical alliance in Shanghai: implications for workforce well-being and health-system management

**DOI:** 10.3389/fpubh.2026.1903738

**Published:** 2026-09-04

**Authors:** Pei Xu, Yanmei Gu, Haonan Shi, Zhendong Zhang, Bei Tian, Tingting Wang

**Affiliations:** 1 Department of Nursing, Zhoupu Hospital Affiliated to Shanghai University of Medicine and Health Sciences, Shanghai, China; 2 School of Medicine, Shanghai University, Shanghai, China; 3 Mental Health Center Affiliated to School of Medicine, Shanghai University (Shanghai Hongkou Mental Health Center), Shanghai, China; 4 School of Nursing & Health Management, Shanghai University of Medicine & Health Sciences, Shanghai, China; 5 Department of Nursing, Pudong Gongli Hospital, Shanghai University of Medicine & Health Sciences, Shanghai, China

**Keywords:** anxiety, burnout, depressive symptoms, health-care workers, insomnia, medical alliance, mental health, occupational health

## Abstract

**Background:**

Health-care workers’ mental health is closely related to workforce stability, quality of care, and health-system resilience. In China, medical alliances are a model for integrating tertiary hospitals, secondary hospitals, and community health-service centres, but evidence on staff mental health within such systems remains limited. The primary objective was to describe the distribution and severity of depressive symptoms, anxiety symptoms, perceived stress, insomnia, and burnout dimensions; the secondary, exploratory objective was to examine adjusted associations with selected demographic and occupational characteristics.

**Methods:**

We audited item-level data from an institution-based cross-sectional online survey conducted in 2024 among health-care workers in a Shanghai medical alliance. Exact duplicate exported records were removed. Depression, anxiety, and stress were scored separately using the DASS-21; sleep symptoms were scored with the ISI; and MBI-GS exhaustion, cynicism, and professional efficacy were analysed as separate dimensions. Internal consistency, multiplicity-adjusted subgroup comparisons, and indicator-coded multivariable models were evaluated.

**Results:**

The supplied file contained 669 rows; 175 were literal copies of earlier records, leaving 494 unique respondents. Descriptively, mean (SD) scores were 9.53 (8.61) for depression, 8.81 (7.95) for anxiety, 11.84 (8.71) for stress, and 9.12 (6.16) for ISI. Scores at or above the mild-symptom threshold occurred in 47.6, 48.6, and 29.4%, respectively; 51.4% scored above the ISI no-clinically-significant-insomnia range. Mean MBI-GS dimension scores were 2.58 (1.62) for exhaustion, 2.26 (1.41) for cynicism, and 3.65 (1.21) for professional efficacy. Cronbach’s alpha ranged from 0.798 to 0.959. In unadjusted subgroup comparisons, only ISI differences by years of work remained statistically significant after Holm correction across 70 tests. In exploratory adjusted models, on-call work was associated with higher depression, anxiety, and ISI scores, and age was associated with ISI; these estimates did not account for institution-level clustering and should not be interpreted as causal effects.

**Conclusion:**

The results indicate a substantial burden of psychological symptoms and measurable burnout dimensions in this medical alliance. Most unadjusted subgroup differences did not withstand multiplicity correction, whereas only a small number of associations were observed in exploratory adjusted models. These findings are non-causal and require confirmation with institution-clustered analyses before being used to define target groups.

## 1 Introduction

The mental health of health-care workers has become a persistent concern for health systems, extending well beyond the acute phase of the COVID-19 pandemic. Clinical work exposes staff to sustained workload, emotional demands, shift work, responsibility for patient safety, and frequent contact with illness, distress, and death. These pressures may be reflected in several psychological outcomes reported within the same workforce, including depressive symptoms, anxiety, perceived stress, sleep disturbance, and burnout; their within-person co-occurrence and directionality require specific analysis. A large systematic review including more than 290,000 medical staff from 46 countries estimated pooled prevalences of burnout, anxiety, depression, insomnia, and stress of 43.6, 37.1, 37.6, 43.7, and 41.3%, respectively (1). An umbrella review of 72 meta-analyses reached a similar conclusion, showing that psychological distress and sleep disturbance among health-care professionals remained highly prevalent during the pandemic and should be understood as a workforce and health-system issue, rather than only as a matter of individual vulnerability (2). Longitudinal evidence further indicates that these outcomes do not necessarily change in parallel: over 14 months in an Italian health-care-worker cohort, mean depression and anxiety scores decreased whereas the mean insomnia score increased (3).

Evidence from China and other countries has shown that psychological distress among health-care workers is shaped by both personal and occupational factors. In Shanghai, medical staff surveyed during the COVID-19 epidemic reported substantial levels of burnout, depression, anxiety, and insomnia, suggesting that large urban health-care systems may place workers under particular psychological strain (4). More recent evidence from China’s post-pandemic period also indicates that depressive and anxiety symptoms remain common among primary health-care workers, especially among women, nurses, workers with longer working hours, and those with less favourable family or occupational conditions (5). Studies using network approaches have further suggested that depression, anxiety, stress, and burnout are closely interconnected, with some symptoms acting as bridges between emotional distress and occupational exhaustion (6). Intervention evidence is also becoming clearer: programmes focused only on individual coping are unlikely to be sufficient unless they are accompanied by organisational measures that address workload, staffing, scheduling, communication, and managerial support (7).

However, several important gaps remain. Many existing studies have focused on single hospitals, frontline staff, or one professional group, particularly doctors and nurses. Fewer have examined the whole workforce within an integrated medical alliance, where tertiary hospitals, secondary hospitals, and community health-service centres are linked through referral, resource sharing, and coordinated service delivery. This organisational structure is increasingly important in China’s health-system reform, which aims to strengthen primary care, improve hierarchical diagnosis and treatment, and promote continuity of care across institutions (8, 9). These reforms may change the distribution of work pressure across institution levels and staff groups, yet the mental health implications for medical-alliance workers have not been adequately described. In addition, these outcomes are often studied separately, leaving limited evidence that describes their concurrent burden and distribution across departments, professional roles, and work schedules within the same health-care network.

To address these gaps, we conducted a cross-sectional survey among health-care workers in a medical alliance in Shanghai, comprising one tertiary general hospital, two secondary specialist hospitals, and five community health-service centres. The primary objective was to estimate the distribution and severity of depressive, anxiety, stress, and insomnia symptoms and the three dimensions of occupational burnout. The secondary, explicitly exploratory objective was to assess adjusted associations with selected demographic and occupational characteristics. Subgroup comparisons were descriptive and were not interpreted as independent effects unless supported by the adjusted models. The study did not test causal effects or formally analyse within-person co-occurrence, correlations, mediation, or other interrelationships among the psychological outcomes.

## 2 Methods

### 2.1 Study design and setting

We conducted a cross-sectional survey among health-care workers employed by an integrated medical consortium in Shanghai, China. The consortium comprised one tertiary general hospital, two secondary specialised hospitals, and five community health centres. Exported submission timestamps ranged from June 13 to August 9, 2024.

The study was designed to describe self-reported mental health and occupational well-being across institutional and occupational settings within the participating medical alliance. Because the design was cross-sectional, all associations were interpreted as non-causal.

### 2.2 Participants and sampling

The eight constituent institutions of the medical alliance were treated as the natural sampling clusters. All eight institutions were included, and a census-style invitation was used: all 825 staff meeting the eligibility criteria were approached. Eligible participants were physicians, nurses, medical technicians, administrative staff, and logistics personnel who had worked in their current post for at least 1 year. Staff were excluded if they had been on leave for more than 3 consecutive months before the survey or had received an administrative disciplinary sanction. The latter criterion was specified *a priori* and was intended to reduce confounding by acute distress directly attributable to an exceptional personnel action rather than routine occupational conditions. We nevertheless recognise that it may have excluded workers with elevated distress and thereby biased prevalence estimates downward.

Because recruitment sought to approach the finite workforce of the participating medical alliance, sample-size adequacy for the primary prevalence-estimation objective was evaluated using the WHO single-proportion approach (10, 11). The initial sample size was *n* = *Z*^2^*P*(1−*P*)/*e*^2^. With *Z* = 1.96, a conservative expected prevalence *p* = 0.50, and an absolute precision *e* = 0.05, *n*0 was 384.16. Because the target population was finite (*N* = 825) and the initial sample exceeded 10% of that population, the finite-population correction *nF* = *n*0/[1 + (*n*0−1)/*N*] gave 262.33. Following WHO guidance for a complex design when a prior design-effect estimate is unavailable, *nF* was multiplied by 1.50 and divided by an anticipated response proportion of 0.80, yielding 491.86, rounded up to a minimum target of 492 participants. The 494 unique analytic records met this precision-based target. This calculation addresses overall prevalence precision; it does not guarantee adequate precision for small subgroups or replace empirical adjustment for institution-level clustering.

### 2.3 Procedures and ethical considerations

Participants completed an anonymous electronic questionnaire after reading the study information. Before accessing the questionnaire, they were required to indicate electronically that they understood the information provided and voluntarily agreed to participate; only respondents who selected the consent option could proceed. This electronically recorded agreement constituted informed consent for the survey. Participation was voluntary, and respondents could withdraw before submission without any effect on professional appraisal, employment assessment, or workplace rights.

The questionnaire was pilot tested before formal administration. Of the 721 returned questionnaires, 52 were excluded during the initial administrative quality-control process: 24 because the recorded completion time was less than 5 min and 28 because the respondent’s actual work did not fall within the scope of this study. This process produced 669 questionnaires classified as valid. During the subsequent item-level audit, rows were treated as literal duplicates only when all exported metadata and responses were identical; 175 duplicate copies were removed, leaving 494 unique records. Scale items had no missing values. The platform’s exported overall total was not analysed because it did not reconcile with the prespecified scale totals; all outcomes were reconstructed directly from their items. One logically inconsistent work-duration value was treated as missing only in analyses involving years of work. Four fully invariant 44-item response patterns were retained because no separate invariant-response exclusion rule was documented.

### 2.4 Measures

#### 2.4.1 Sociodemographic and occupational characteristics

A self-designed general information form was used to collect sex, age, marital status, educational attainment, occupational role, professional title, years of work experience, department, work schedule, and the level of the employing medical institution.

#### 2.4.2 Depression, anxiety, and stress

Negative emotional symptoms were assessed with the Chinese version of the 21-item Depression Anxiety Stress Scale (DASS-21) (12–14). Each item is scored from 0 to 3. Depression (items 3, 5, 10, 13, 16, 17, and 21), anxiety (items 2, 4, 7, 9, 15, 19, and 20), and stress (items 1, 6, 8, 11, 12, 14, and 18) raw sums were multiplied by two. Conventional descriptive bands were normal/mild/moderate/severe/extremely severe at 0–9/10–13/14–20/21–27/28 or higher for depression; 0–7/8–9/10–14/15–19/20 or higher for anxiety; and 0–14/15–18/19–25/26–33/34 or higher for stress. These categories were not interpreted as clinical diagnoses. In this sample, Cronbach’s alpha (bootstrap 95% CI) was 0.889 (0.868–0.906) for depression, 0.869 (0.841–0.891) for anxiety, 0.873 (0.849–0.892) for stress, and 0.953 (0.943–0.960) for all 21 items.

#### 2.4.3 Insomnia

Sleep problems were assessed with the Chinese version of the seven-item Insomnia Severity Index (ISI) (15). Items are summed to give a score from 0 to 28. Scores were described as no clinically significant insomnia (0–7), subthreshold insomnia (8–14), moderate clinical insomnia (15–21), and severe clinical insomnia (22–28); these are symptom-severity bands, not clinical diagnoses. Cronbach’s alpha was 0.947 (bootstrap 95% CI 0.938–0.954).

#### 2.4.4 Occupational burnout

Occupational burnout was assessed with the 16-item MBI-GS form present in the survey export (16). The administered form retained five cynicism items, unlike the 15-item Chinese revision that deleted one cynicism item (17). Items are rated from 0 (never) to 6 (daily). Dimension scores were calculated as item means: exhaustion used items 1, 2, 3, 4, and 6; cynicism used items 8, 9, 12, 13, and 14; and professional efficacy used items 5, 7, 10, 11, 15, and 16. Because item 16 was negatively worded, it was reverse-coded as 6 minus the recorded response before the professional-efficacy mean was calculated. Higher exhaustion and cynicism and lower professional efficacy indicate a less favourable burnout profile. The dimensions were not combined, and the previous low/severe/very severe categories were withdrawn because the MBI is not a diagnostic instrument and current guidance does not support those cut-offs. Cronbach’s alpha was 0.959 (95% CI 0.952–0.966) for exhaustion, 0.869 (0.847–0.889) for cynicism, and 0.798 (0.767–0.825) for professional efficacy.

### 2.5 Statistical analysis

Categorical variables were summarised as counts and percentages and continuous variables as means and standard deviations. Reliability was estimated using Cronbach’s alpha with 2,000-resample percentile bootstrap 95% CIs. Two-category subgroup comparisons used Welch *t* tests with Hedges’ *g*; multi-category comparisons used Welch one-way ANOVA with descriptive eta-squared. The 70 omnibus *p* values (ten characteristics by seven outcomes) were adjusted together using the Holm method. For each multi-category factor with an unadjusted omnibus *p* value below 0.05, all pairwise Welch contrasts were reported with Holm adjustment within that factor-outcome family.

For provisional adjusted analyses, separate ordinary least-squares models were fitted for DASS-21 depression, anxiety, stress, ISI, MBI-GS exhaustion, cynicism, and professional efficacy. Nominal variables were represented by indicator terms. Reference groups were community health-service centre, administrative staff, male sex, and day shift; age was centred and scaled per 10 years. The same *a priori* predictors were used for all outcomes: institution level, occupation, sex, age, and shift pattern. Age was retained instead of years of work because their correlation was *r* = 0.957. Occupation was retained instead of department because their association was strong (Cramer’s *V* = 0.696); entering both produced variance inflation factors above 10. Education and professional title were not included because of sparse or structurally overlapping categories, and covariates were not selected by univariable *p* values. HC3 heteroskedasticity-robust standard errors were used. Model fit was summarised by *R*-squared and adjusted *R*-squared; VIFs, condition numbers, studentised residuals, Cook’s distances, and Breusch-Pagan tests were examined. Because the analysis export contained institution level but not identifiers for the eight individual institutions, institution-level ICCs and cluster-adjusted standard errors could not be estimated. The models were therefore treated as exploratory individual-level analyses, and this limitation was considered when interpreting their confidence intervals.

## 3 Results

### 3.1 Questionnaire return and participant characteristics

Of the 825 eligible workers approached, 721 returned a questionnaire, giving an actual questionnaire return rate of 87.4% (721/825). After the initial exclusion of 52 returned questionnaires (24 completed in less than 5 min and 28 submitted by workers outside the study scope), 669 were administratively classified as valid. The valid-return proportion was 92.8% (669/721), and the nominal valid-response proportion among those approached was 81.1% (669/825). Audit of the supplied workbook identified 175 literal duplicate copies, all located in appended rows 495–669, leaving 494 unique analytic records, equivalent to 59.9% of the workers approached. The duplicate rows were treated as an export/data-assembly issue and were not counted as additional respondents.

The mean age of the 494 unique respondents was 38.39 years (SD 8.56), and mean work duration was 16.11 years (SD 9.53; *n* = 493). Most respondents worked in the tertiary hospital [258 (52.2%)], were women [414 (83.8%)], and held nursing posts [212 (42.9%); Table 1].

**Table 1:** Participant characteristics (*n* = 494).

Characteristic	Category	*n*	%
Hospital type	Tertiary hospital	258	52.2
	Secondary hospital	96	19.4
	Community health-service centre	140	28.3
Job category	Physician	109	22.1
	Nurse	212	42.9
	Medical technician	107	21.7
	Logistics staff	23	4.7
	Administrative staff	43	8.7
Department	Internal medicine	87	17.6
	Surgery	15	3.0
	Obstetrics and gynaecology	15	3.0
	Outpatient department	57	11.5
	Emergency department	38	7.7
	Operating room	17	3.4
	Intensive care unit	17	3.4
	General department	24	4.9
	Laboratory or examination department	63	12.8
	Administrative department	46	9.3
	Logistics department	14	2.8
	Other	101	20.4
Sex	Male	80	16.2
	Female	414	83.8
Age, years	20–30	109	22.1
	31–40	195	39.5
	41–50	145	29.4
	>50	45	9.1
Marital status	Unmarried	86	17.4
	Married	390	78.9
	Divorced	18	3.6
Education	High school or technical secondary school	3	0.6
	Junior college	79	16.0
	Bachelor degree	385	77.9
	Postgraduate degree or above	27	5.5
Professional title	Junior	193	39.1
	Intermediate	220	44.5
	Senior	41	8.3
	No professional title	40	8.1
Years of work	<=2	23	4.7
	3–5	48	9.7
	6–10	98	19.8
	11–20	174	35.2
	>20	150	30.4
	Missing	1	0.2
Shift pattern	Day shift	285	57.7
	On-call shift	101	20.4
	Evening or night shift	108	21.9

Percentages use 494 as the denominator. One implausible work-duration value was set to missing, so work-duration percentages include a missing category. Department frequencies, subgroup comparisons, and regression-coding decisions were recalculated directly from the numeric codes in the supplied raw dataset.

### 3.2 Negative emotional symptoms

The mean DASS-21 depression score was 9.53 (SD 8.61), and 235 (47.6%) respondents scored at or above the mild-symptom threshold. The mean anxiety score was 8.81 (SD 7.95), with 240 (48.6%) at or above the mild threshold. The mean stress score was 11.84 (SD 8.71), and 145 (29.4%) scored at or above the mild threshold (Table 2).

**Table 2:** Negative emotional symptoms assessed using DASS-21.

Severity	Depression, *n* (%)	Anxiety, *n* (%)	Stress, *n* (%)
Normal	259 (52.4%)	254 (51.4%)	349 (70.6%)
Mild	99 (20.0%)	54 (10.9%)	64 (13.0%)
Moderate	85 (17.2%)	98 (19.8%)	37 (7.5%)
Severe	20 (4.0%)	40 (8.1%)	32 (6.5%)
Extremely severe	31 (6.3%)	48 (9.7%)	12 (2.4%)
Mean (SD)	9.53 (8.61)	8.81 (7.95)	11.84 (8.71)

DASS-21, Depression Anxiety Stress Scales-21. Values are *n* (%) unless otherwise indicated.

### 3.3 Insomnia symptoms

The mean ISI score was 9.12 (SD 6.16). Overall, 254 (51.4%) respondents scored above the no-clinically-significant-insomnia range: 172 (34.8%) had subthreshold insomnia, 54 (10.9%) moderate clinical insomnia, and 28 (5.7%) severe clinical insomnia (Table 3).

**Table 3:** Insomnia severity assessed using the ISI.

ISI severity	*n*	%
No clinically significant insomnia	240	48.6
Subthreshold insomnia	172	34.8
Moderate clinical insomnia	54	10.9
Severe clinical insomnia	28	5.7
Mean (SD)	9.12 (6.16)	

ISI, Insomnia Severity Index.

### 3.4 Occupational burnout

Mean (SD) dimension scores were 2.58 (1.62) for exhaustion, 2.26 (1.41) for cynicism, and 3.65 (1.21) for professional efficacy (Table 4). These continuous dimensions are not directly comparable with the DASS-21 or ISI symptom thresholds and were not converted into diagnostic severity categories.

**Table 4:** MBI-GS dimension scores (n = 494).

Dimension	Mean (SD)	Cronbach’s alpha (95% CI)
Exhaustion	2.58 (1.62)	0.959 (0.952–0.966)
Cynicism	2.26 (1.41)	0.869 (0.847–0.889)
Professional efficacy	3.65 (1.21)	0.798 (0.767–0.825)

MBI-GS, Maslach Burnout Inventory-General Survey. Scores are item means on a 0–6 scale. Higher exhaustion and cynicism and lower professional efficacy indicate a less favourable profile. Alpha CIs are percentile bootstrap CIs based on 2,000 resamples.

### 3.5 Differences in mental health indicators across participant subgroups

Unadjusted subgroup comparisons were recalculated for all seven outcomes (Table 5; Supplementary Tables S1, S2). Before family-wide correction, several factors had *p* values below 0.05, but after Holm adjustment across all 70 omnibus tests, only ISI by years of work remained statistically significant (Holm-adjusted *p* = 0.007; eta-squared = 0.037). ISI means were 5.48 (SD 4.42) for 2 years or less, 7.79 (5.06) for 6–10 years, 9.67 (6.32) for 11–20 years, and 10.07 (6.48) for more than 20 years. Holm-adjusted pairwise differences were 4.60 (95% CI 2.44–6.75) for >20 versus <=2 years, 4.19 (2.08–6.30) for 11–20 versus <=2 years, and 2.29 (0.84–3.74) for >20 versus 6–10 years.

**Table 5:** Univariable omnibus comparisons of psychological outcomes.

Characteristic	Dep.	Anx.	Stress	ISI	Exh.	Cyn.	PE
Hospital type	0.351/1.000; eta2 = 0.004	0.568/1.000; eta2 = 0.002	0.367/1.000; eta2 = 0.004	0.047/1.000; eta2 = 0.012	0.090/1.000; eta2 = 0.010	0.013/0.730; eta2 = 0.019	0.431/1.000; eta2 = 0.003
Job category	0.088/1.000; eta2 = 0.019	0.615/1.000; eta2 = 0.006	0.149/1.000; eta2 = 0.015	0.096/1.000; eta2 = 0.020	0.012/0.726; eta2 = 0.030	0.003/0.198; eta2 = 0.041	0.765/1.000; eta2 = 0.004
Department	0.610/1.000; eta2 = 0.011	0.353/1.000; eta2 = 0.026	0.850/1.000; eta2 = 0.013	0.233/1.000; eta2 = 0.031	0.930/1.000; eta2 = 0.010	0.034/1.000; eta2 = 0.025	0.468/1.000; eta2 = 0.022
Sex	0.032/1.000; *g* = −0.315	0.373/1.000; *g* = −0.129	0.017/0.900; *g* = −0.371	0.114/1.000; *g* = −0.242	0.105/1.000; *g* = −0.220	0.056/1.000; *g* = −0.251	0.187/1.000; *g* = 0.182
Age group	0.018/0.940; eta2 = 0.020	0.013/0.730; eta2 = 0.022	0.006/0.414; eta2 = 0.027	0.012/0.730; eta2 = 0.023	0.008/0.489; eta2 = 0.021	0.012/0.730; eta2 = 0.022	0.024/1.000; eta2 = 0.021
Marital status	0.640/1.000; eta2 = 0.003	0.788/1.000; eta2 = 0.001	0.811/1.000; eta2 = 0.001	0.070/1.000; eta2 = 0.011	0.792/1.000; eta2 = 0.001	0.574/1.000; eta2 = 0.003	0.405/1.000; eta2 = 0.004
Education	0.271/1.000; eta2 = 0.009	0.060/1.000; eta2 = 0.006	0.202/1.000; eta2 = 0.010	0.378/1.000; eta2 = 0.007	0.018/0.962; eta2 = 0.030	0.133/1.000; eta2 = 0.015	0.739/1.000; eta2 = 0.003
Professional title	0.479/1.000; eta2 = 0.005	0.154/1.000; eta2 = 0.008	0.178/1.000; eta2 = 0.010	0.002/0.163; eta2 = 0.028	0.262/1.000; eta2 = 0.008	0.328/1.000; eta2 = 0.006	0.217/1.000; eta2 = 0.010
Years of work	0.096/1.000; eta2 = 0.011	0.664/1.000; eta2 = 0.005	0.371/1.000; eta2 = 0.008	<0.001/0.007; eta2 = 0.037	0.963/1.000; eta2 = 0.001	0.773/1.000; eta2 = 0.004	0.016/0.858; eta2 = 0.026
Shift pattern	0.003/0.223; eta2 = 0.028	0.009/0.573; eta2 = 0.020	0.022/1.000; eta2 = 0.018	0.001/0.071; eta2 = 0.032	0.012/0.726; eta2 = 0.019	0.014/0.767; eta2 = 0.020	0.036/1.000; eta2 = 0.013

Cells show unadjusted *p*/Holm-adjusted *p* and effect size. Welch one-way ANOVA with eta-squared was used for multi-category factors; Welch *t* test with Hedges’ *g* was used for sex. Holm adjustment covered all 70 omnibus tests. Complete group means and all Holm-adjusted post-hoc contrasts for factors with unadjusted omnibus *p* < 0.05 are in Supplementary Tables S1, S2.

### 3.6 Multivariable linear regression analyses

In provisional indicator-coded models, shift pattern was associated jointly with depression (Wald *p* = 0.004) and anxiety (*p* = 0.009), but not stress (*p* = 0.072). Compared with day shift, on-call shift was associated with higher depression (*B* = 3.42, 95% CI 1.20–5.65), anxiety (*B* = 2.59, 0.67–4.50), and stress (*B* = 2.43, 0.31–4.55) scores. Evening/night shift was associated with higher depression (*B* = 2.23, 0.05–4.41) and anxiety (*B* = 2.22, 0.17–4.28). Female sex was associated with a lower stress score (*B* = −2.77, −5.49 to −0.04). These estimates are exploratory and uncorrected across model coefficients (Table 6).

**Table 6:** Provisional HC3 linear models for DASS-21 subscale scores.

Predictor	Depression *B* [95% CI]; *p*	Anxiety *B* [95% CI]; *p*	Stress *B* [95% CI]; *p*
Hospital: secondary vs. community	−0.58 [−3.06, 1.89]; *p* = 0.643	−0.53 [−2.72, 1.66]; *p* = 0.634	−1.20 [−3.68, 1.28]; *p* = 0.342
Hospital: tertiary vs. community	−0.92 [−2.78, 0.94]; *p* = 0.333	−0.92 [−2.57, 0.72]; *p* = 0.271	−0.78 [−2.70, 1.14]; *p* = 0.427
Logistics vs. administrative staff	−0.00 [−4.52, 4.52]; *p* = 1.000	0.43 [−3.50, 4.36]; *p* = 0.830	0.87 [−3.81, 5.56]; *p* = 0.715
Medical technician vs. administrative staff	−1.25 [−4.23, 1.74]; *p* = 0.413	−0.73 [−3.68, 2.22]; *p* = 0.627	−0.12 [−3.29, 3.05]; *p* = 0.939
Nurse vs. administrative staff	−1.89 [−4.56, 0.79]; *p* = 0.167	−0.35 [−2.94, 2.23]; *p* = 0.789	−0.12 [−2.95, 2.72]; *p* = 0.936
Physician vs. administrative staff	−0.56 [−3.69, 2.58]; *p* = 0.727	−0.11 [−2.97, 2.76]; *p* = 0.943	0.77 [−2.45, 4.00]; *p* = 0.639
Female vs. male	−1.97 [−4.61, 0.68]; *p* = 0.145	−0.90 [−3.34, 1.53]; *p* = 0.467	−2.77 [−5.49, −0.04]; *p* = 0.046
Evening/night vs. day shift	2.23 [0.05, 4.41]; *p* = 0.045	2.22 [0.17, 4.28]; *p* = 0.034	1.10 [−1.06, 3.26]; *p* = 0.318
On-call vs. day shift	3.42 [1.20, 5.65]; *p* = 0.003	2.59 [0.67, 4.50]; *p* = 0.008	2.43 [0.31, 4.55]; *p* = 0.025
Age, per 10 years	0.40 [−0.50, 1.30]; *p* = 0.384	0.12 [−0.72, 0.95]; *p* = 0.786	0.31 [−0.63, 1.25]; *p* = 0.519
*R*^2^/adjusted *R*^2^	0.049/0.029	0.027/0.007	0.039/0.019

*n* = 494. *B* values are unstandardised coefficients. Reference categories: community health-service centre, administrative staff, male, and day shift. HC3 standard errors address heteroskedasticity but not institution-level clustering; the coefficients and confidence intervals should therefore be interpreted as exploratory individual-level estimates.

For ISI, shift pattern was jointly associated with score (Wald *p* = 0.002). On-call workers scored 2.69 points higher than day-shift workers (95% CI 1.16–4.22; *p* < 0.001). Each additional 10 years of age was associated with a 1.09-point higher ISI score (0.45–1.73; *p* < 0.001). Institution level, occupation, and sex were not jointly associated with ISI in this provisional model (Table 7).

**Table 7:** Provisional HC3 linear model for ISI score.

Predictor	*B*	95% CI	*p*
Hospital: secondary vs. community	−0.51	−2.36 to 1.33	0.584
Hospital: tertiary vs. community	−1.28	−2.65 to 0.10	0.069
Logistics vs. administrative staff	−1.76	−4.72 to 1.21	0.246
Medical technician vs. administrative staff	−0.57	−2.86 to 1.73	0.629
Nurse vs. administrative staff	0.34	−1.81 to 2.48	0.759
Physician vs. administrative staff	−0.07	−2.54 to 2.41	0.958
Female vs. male	−1.21	−3.16 to 0.74	0.223
Evening/night vs. day shift	1.07	−0.36 to 2.49	0.143
On-call vs. day shift	2.69	1.16 to 4.22	<0.001
Age, per 10 years	1.09	0.45 to 1.73	<0.001
*R*^2^/adjusted *R*^2^	0.070	0.050	

*n* = 494. Reference categories and analytical limitations are the same as Table 6.

No categorical factor was jointly associated with exhaustion, cynicism, or professional efficacy in the provisional models. Each additional 10 years of age was associated with a 0.20-point higher professional-efficacy score (95% CI 0.06–0.34; *p* = 0.004), whereas its associations with exhaustion and cynicism were near zero. Model *R*-squared values were low (0.042–0.057; Table 8).

**Table 8:** Provisional HC3 linear models for MBI-GS dimensions.

Predictor	Exhaustion *B* [95% CI]; *p*	Cynicism *B* [95% CI]; *p*	Professional efficacy *B* [95% CI]; *p*
Hospital: secondary vs. community	−0.24 [−0.68, 0.20]; *p* = 0.281	−0.39 [−0.79, 0.01]; *p* = 0.054	0.20 [−0.11, 0.52]; *p* = 0.204
Hospital: tertiary vs. community	−0.30 [−0.66, 0.06]; *p* = 0.105	−0.32 [−0.65, 0.00]; *p* = 0.053	0.26 [−0.02, 0.54]; *p* = 0.070
Logistics vs. administrative staff	−0.06 [−0.92, 0.80]; *p* = 0.894	−0.18 [−0.86, 0.50]; *p* = 0.601	0.32 [−0.33, 0.97]; *p* = 0.332
Medical technician vs. administrative staff	−0.12 [−0.70, 0.46]; *p* = 0.692	−0.04 [−0.50, 0.42]; *p* = 0.863	0.13 [−0.28, 0.53]; *p* = 0.534
Nurse vs. administrative staff	−0.09 [−0.62, 0.45]; *p* = 0.746	−0.03 [−0.45, 0.40]; *p* = 0.898	0.06 [−0.32, 0.44]; *p* = 0.753
Physician vs. administrative staff	0.40 [−0.22, 1.02]; *p* = 0.204	0.43 [−0.07, 0.93]; *p* = 0.094	0.12 [−0.33, 0.56]; *p* = 0.604
Female vs. male	−0.25 [−0.71, 0.20]; *p* = 0.280	−0.24 [−0.62, 0.14]; *p* = 0.213	0.27 [−0.07, 0.61]; *p* = 0.119
Evening/night vs. day shift	0.36 [−0.06, 0.77]; *p* = 0.093	0.11 [−0.22, 0.45]; *p* = 0.517	−0.12 [−0.43, 0.19]; *p* = 0.439
On-call vs. day shift	0.35 [−0.06, 0.75]; *p* = 0.092	0.26 [−0.09, 0.61]; *p* = 0.150	−0.28 [−0.57, 0.02]; *p* = 0.066
Age, per 10 years	−0.02 [−0.20, 0.15]; *p* = 0.784	−0.01 [−0.16, 0.14]; *p* = 0.936	0.20 [0.06, 0.34]; *p* = 0.004
*R*^2^/adjusted *R*^2^	0.046/0.026	0.057/0.038	0.042/0.022

*n* = 494. Higher professional efficacy is favourable. Across models, maximum VIF was 3.83 and the condition number was 13.33. Breusch–Pagan tests suggested heteroskedasticity for depression, stress, and ISI, supporting HC3 inference. There were 23–33 observations per model with Cook’s *D* > 4/*n*, indicating that influence diagnostics also warrant cautious interpretation. Institution-level ICCs and cluster-adjusted standard errors could not be estimated from the available export, so all model-based inferences remain exploratory.

## 4 Discussion

After removal of literal duplicate records and reconstruction of scores from individual items, this cross-sectional survey found that 47.6% of respondents scored at or above the mild threshold for depression, 48.6% for anxiety, 29.4% for stress, and 51.4% above the no-clinically-significant-insomnia range. These bands describe self-reported symptom severity and are not clinical diagnoses. Burnout was represented by mean exhaustion, cynicism, and professional-efficacy scores rather than an unsupported global severity classification.

The recalculated depressive and anxiety symptom proportions remain substantial. Chinese studies conducted during the late-pandemic surge, post-pandemic period, and policy reopening likewise reported appreciable depressive, anxiety, stress, or insomnia symptoms, although estimates varied by setting, instrument, and period (18–21). The stress proportion was substantially lower than the original report because the standard seven-item stress key and conventional doubled subscale score were applied. The revised values should therefore replace the previously reported global or incompatible DASS results.

The large number of subgroup comparisons produced several nominal *p* values below 0.05, but only the ISI difference across work-duration groups survived Holm adjustment across the 70 omnibus tests. This pattern underscores why raw subgroup *p* values should not be interpreted as established differences. The effect size was small (eta-squared = 0.037), and the cross-sectional design cannot distinguish cumulative occupational exposure from age, cohort, or survivorship effects.

Sex-related findings require cautious interpretation. Women comprised 83.8% of the unique sample and were disproportionately represented in nursing and other patient-facing roles. Female sex was associated with a lower stress score in the provisional model, but the confidence interval barely excluded zero and institution clustering was unavailable. Sex was strongly patterned by occupation and may remain confounded by department, schedule, and unmeasured family responsibilities; the coefficient therefore does not establish that female sex itself is protective.

Sleep disturbance was prominent: 51.4% of respondents scored above the ISI no-clinically-significant-insomnia range. In the provisional adjusted model, older age and on-call work were associated with higher ISI scores, whereas rotating shift was not. Accordingly, the descriptive sleep patterns should not be presented as evidence of an independent rotating-shift effect. External studies nevertheless link insomnia with resilience and negative emotional symptoms among nurses, and reviews and structural models identify night work and occupational stress as plausible correlates of sleep disturbance (22–24). These external associations provide context but do not overturn the null rotating-shift coefficient in this study. The age and on-call coefficients are compatible with differences in sleep opportunity and recovery, but the cross-sectional design does not establish directionality, and the provisional confidence intervals should be interpreted cautiously because institution-level clustering could not be incorporated.

Sleep disturbance is relevant to workforce well-being because it may impair recovery and co-occur with emotional symptoms and burnout. A Chinese nurse study reported associations among insomnia, burnout, anxiety, and depression, while a systematic review examined sleep interventions for rotating night-shift workers (25, 26). The present survey did not test mediation or intervention effects. Accordingly, these external findings provide context for potential sleep-health support but do not demonstrate that sleep disturbance caused burnout in this sample or that a particular intervention would be effective in the participating institutions.

Occupational burnout was treated as a multidimensional continuum. Mean exhaustion and cynicism scores were 2.58 and 2.26 on the 0–6 metric, while mean professional efficacy was 3.65. The lower prevalence of severe burnout cited in the original report cannot be compared with DASS-21 or ISI proportions because the former categories were unsupported and the constructs use different scales, timeframes, and thresholds. Separate dimensions avoid the misleading implication that a person either has or does not have a single burnout diagnosis. Studies across health-care settings have linked burnout with work overload, and a recent meta-analysis associated nurse burnout with poorer patient safety, satisfaction, and quality of care (27, 28). These external findings establish the organisational relevance of burnout but do not validate a global diagnostic cut-off for the MBI-GS.

The medical-alliance setting remains organisationally relevant because integrated-care pathways and provider experiences can be shaped by cross-institutional coordination; prior Chinese studies have examined continuity of care and provider satisfaction within medical alliances (29, 30). A recent scoping review reported broadly consistent associations between lower job satisfaction and poorer mental health across health-care roles, while noting that the predominantly cross-sectional evidence does not establish directionality (31). Job satisfaction was not measured in this survey and therefore cannot explain the present findings. Moreover, the available export retained institution level rather than the identities of the eight institutions; between-institution heterogeneity and within-institution correlation could not be estimated, limiting institution-level interpretation.

The findings may have practical implications for health-system management, but the organisational recommendations should be viewed as potential responses to the descriptive burden rather than interventions proven effective by this study. Evidence linking work overload with burnout and intent to leave, together with systematic reviews of workplace mental-health interventions, supports considering organisational as well as individual-level responses (32–34). Confidential and non-punitive monitoring, access to psychological and sleep-health support, and review of workload and scheduling may be considered within medical alliances. Any priority groups or specific measures should be based on locally confirmed needs and the exploratory adjusted estimates, not on nominal unadjusted subgroup differences alone.

This study included health-care workers from a tertiary general hospital, secondary specialist hospitals, and community health-service centres and covered physicians, nurses, medical technicians, administrative staff, and logistics personnel. It also assessed depressive, anxiety, and stress symptoms, insomnia, and occupational burnout dimensions in the same survey. This breadth allows the burden of several aspects of workforce mental health to be described within one organisational network, although their co-occurrence and interrelationships were not formally analysed.

Several limitations should be acknowledged. The cross-sectional design precludes causal inference. The study was conducted in one Shanghai medical alliance and used self-reported symptom scales rather than diagnostic interviews. Although the questionnaire return rate was 87.4%, demographic and occupational information was unavailable for the 104 invited workers who did not return a questionnaire and the 52 administratively excluded returns. Non-response and exclusion bias therefore could not be quantified. If workers experiencing greater distress were either more motivated to participate or less able or willing to respond, the prevalence estimates could, respectively, be biased upward or downward. The administrative-disciplinary-sanction exclusion may also have disproportionately removed workers experiencing distress related to personnel actions and thus could have biased prevalence estimates downward; the available analytic export did not permit this effect to be quantified. The source file also contained 175 literal duplicate rows; although these copies were removed, the discrepancy between the reported exclusion of 24 responses completed in less than 5 min and the completion-time distribution in the supplied workbook requires resolution. The original 721-row export and contemporaneous exclusion log should be retained to permit a complete audit. Institution identity was absent, so provisional models could not account for within-facility correlation and may have confidence intervals that are too narrow. Holm adjustment reduced false-positive risk, but subgroup analyses remained exploratory and several categories were small. One work-duration value was logically inconsistent. Finally, unmeasured workload, night-shift frequency, patient volume, workplace violence, work–family conflict, social support, and mental-health history may have produced residual confounding.

## 5 Conclusion

Among 494 unique respondents working across an integrated medical alliance, the descriptive results showed substantial depressive and anxiety symptoms, perceived stress and insomnia symptoms, together with measurable burnout dimensions. Most unadjusted subgroup differences were attenuated after multiplicity correction, and only a small number of associations were observed in the provisional multivariable models. The results support considering alliance-wide mental-health and sleep-health surveillance and support, but they do not establish that institution level, professional role, department, work experience, sex, or shift pattern are independent causal determinants. Any targeted action should be guided by local confirmation and analyses that can account for clustering within institutions.

## Data Availability

The raw data supporting the conclusions of this article will be made available by the authors, without undue reservation.
