# Supplementary material for: Depressive symptoms, insomnia, and burnout among health-care workers in an integrated medical alliance in Shanghai: implications for workforce well-being and health-system management
Source: Front Public Health. 2026 Sep 4;14:1903738. doi: 10.3389/fpubh.2026.1903738 (PMC13585710; doi:10.3389/fpubh.2026.1903738)
Supplement: Supplementary file 1 [file Table_1.docx]

**Supplementary Table S1. Holm-adjusted post-hoc contrasts for multi-category factors with unadjusted omnibus p<0.05**

| Factor | Outcome | Contrast | Mean diff. | 95% CI | Holm p |
| --- | --- | --- | --- | --- | --- |
| Hospital type | Cynicism | Tertiary hospital vs Community health-service centre | -0.44 | -0.74 to -0.14 | 0.012 |
| Job category | Exhaustion | Nurse vs Physician | -0.66 | -1.05 to -0.28 | 0.008 |
| Job category | Exhaustion | Physician vs Medical technician | 0.71 | 0.26 to 1.16 | 0.017 |
| Job category | Cynicism | Nurse vs Physician | -0.70 | -1.04 to -0.35 | 0.001 |
| Job category | Cynicism | Physician vs Medical technician | 0.68 | 0.28 to 1.08 | 0.009 |
| Department | Cynicism | Outpatient department vs Operating room | 0.94 | 0.42 to 1.45 | 0.038 |
| Age group | Anxiety | 31-40 vs >50 | 3.52 | 0.97 to 6.08 | 0.046 |
| Age group | Stress | 41-50 vs 20-30 | 3.19 | 0.98 to 5.39 | 0.029 |
| Age group | Stress | 20-30 vs 31-40 | -2.88 | -4.90 to -0.86 | 0.029 |
| Age group | ISI | 41-50 vs 20-30 | 2.64 | 1.08 to 4.19 | 0.006 |
| Age group | Exhaustion | 41-50 vs >50 | 0.84 | 0.36 to 1.33 | 0.005 |
| Age group | Cynicism | 41-50 vs >50 | 0.69 | 0.26 to 1.13 | 0.012 |
| Age group | Professional efficacy | 31-40 vs >50 | -0.60 | -1.01 to -0.19 | 0.029 |
| Education | Exhaustion | Bachelor degree vs Junior college | 0.69 | 0.33 to 1.05 | 0.001 |
| Education | Exhaustion | Postgraduate degree or above vs Junior college | 1.10 | 0.46 to 1.74 | 0.006 |
| Professional title | ISI | Intermediate vs Junior | 1.83 | 0.66 to 3.00 | 0.011 |
| Professional title | ISI | Intermediate vs No professional title | 3.02 | 1.21 to 4.84 | 0.009 |
| Years of work | ISI | >20 vs 6-10 | 2.29 | 0.84 to 3.74 | 0.017 |
| Years of work | ISI | >20 vs <=2 | 4.60 | 2.44 to 6.75 | 0.001 |
| Years of work | ISI | 11-20 vs <=2 | 4.19 | 2.08 to 6.30 | 0.003 |
| Shift pattern | Depression | Day shift vs On-call shift | -3.70 | -5.84 to -1.57 | 0.002 |
| Shift pattern | Anxiety | Day shift vs On-call shift | -2.75 | -4.64 to -0.85 | 0.014 |
| Shift pattern | Stress | Day shift vs On-call shift | -2.92 | -5.02 to -0.82 | 0.020 |
| Shift pattern | ISI | Day shift vs On-call shift | -2.75 | -4.23 to -1.27 | 0.001 |
| Shift pattern | ISI | Evening or night shift vs On-call shift | -2.71 | -4.41 to -1.02 | 0.004 |
| Shift pattern | Exhaustion | Day shift vs On-call shift | -0.57 | -0.94 to -0.20 | 0.009 |
| Shift pattern | Cynicism | Day shift vs On-call shift | -0.47 | -0.81 to -0.13 | 0.021 |
| Shift pattern | Cynicism | Evening or night shift vs On-call shift | -0.54 | -0.94 to -0.14 | 0.021 |
| Shift pattern | Professional efficacy | Day shift vs On-call shift | 0.33 | 0.07 to 0.59 | 0.041 |

**Table S2A. Observed DASS-21 and ISI scores by participant characteristics**

| Characteristic | Category | n | Depression | Anxiety | Stress | ISI |
| --- | --- | --- | --- | --- | --- | --- |
| Hospital type | Tertiary hospital | 258 | 9.03 (8.75) | 8.46 (8.32) | 11.48 (8.91) | 8.48 (5.75) |
|  | Community health-service centre | 140 | 10.26 (7.93) | 9.23 (6.81) | 12.66 (7.69) | 9.96 (6.17) |
|  | Secondary hospital | 96 | 9.83 (9.17) | 9.17 (8.51) | 11.60 (9.55) | 9.60 (7.00) |
| Job category | Nurse | 212 | 8.50 (7.88) | 8.71 (7.44) | 11.04 (7.96) | 8.92 (5.60) |
|  | Physician | 109 | 11.52 (9.76) | 9.85 (8.40) | 13.74 (9.39) | 10.64 (7.12) |
|  | Administrative staff | 43 | 9.81 (7.93) | 8.23 (7.65) | 11.26 (8.54) | 8.91 (6.51) |
|  | Medical technician | 107 | 9.23 (8.84) | 8.11 (8.73) | 11.53 (9.25) | 8.30 (5.95) |
|  | Logistics staff | 23 | 10.52 (8.40) | 9.22 (7.20) | 12.70 (9.10) | 7.87 (5.64) |
| Department | Administrative department | 46 | 9.70 (7.77) | 7.83 (7.42) | 11.57 (8.43) | 8.70 (6.19) |
|  | Surgery | 15 | 7.87 (10.54) | 9.07 (11.18) | 11.60 (11.09) | 9.47 (6.10) |
|  | Outpatient department | 57 | 9.93 (8.33) | 9.72 (7.74) | 12.25 (8.13) | 9.75 (6.31) |
|  | Internal medicine | 87 | 10.23 (9.19) | 9.26 (7.94) | 12.09 (8.64) | 9.83 (6.22) |
|  | Obstetrics and gynaecology | 15 | 7.33 (7.62) | 6.80 (5.99) | 10.53 (6.07) | 10.00 (7.69) |
|  | Laboratory or examination department | 63 | 8.57 (8.47) | 6.63 (7.77) | 9.94 (8.72) | 8.52 (5.78) |
|  | Other | 101 | 9.54 (8.83) | 9.11 (7.91) | 12.34 (8.45) | 7.91 (5.70) |
|  | Operating room | 17 | 6.94 (4.19) | 7.88 (4.97) | 10.71 (5.57) | 8.76 (3.70) |
|  | Emergency department | 38 | 10.47 (9.09) | 10.89 (9.00) | 13.32 (9.48) | 9.71 (6.30) |
|  | Logistics department | 14 | 9.71 (8.55) | 7.86 (7.50) | 10.86 (10.55) | 7.57 (6.07) |
|  | General department | 24 | 10.17 (8.17) | 11.00 (8.49) | 13.33 (9.09) | 12.46 (6.46) |
|  | Intensive care unit | 17 | 10.59 (10.74) | 8.00 (8.34) | 11.88 (12.15) | 8.35 (8.01) |
| Sex | Female | 414 | 9.10 (8.13) | 8.65 (7.57) | 11.32 (8.05) | 8.88 (5.73) |
|  | Male | 80 | 11.80 (10.54) | 9.68 (9.71) | 14.53 (11.22) | 10.36 (7.93) |
| Age group | 41-50 | 145 | 10.63 (8.75) | 9.37 (8.23) | 12.97 (8.94) | 10.30 (7.00) |
|  | 20-30 | 109 | 7.85 (8.63) | 7.58 (7.68) | 9.78 (8.77) | 7.66 (5.56) |
|  | 31-40 | 195 | 10.13 (8.49) | 9.70 (7.78) | 12.66 (8.17) | 9.05 (5.82) |
|  | >50 | 45 | 7.47 (7.92) | 6.18 (7.73) | 9.64 (9.12) | 9.13 (5.42) |
| Marital status | Married | 390 | 9.39 (8.35) | 8.69 (7.92) | 11.83 (8.64) | 9.24 (6.15) |
|  | Unmarried | 86 | 9.74 (9.42) | 9.19 (8.20) | 11.65 (9.25) | 8.03 (6.06) |
|  | Divorced | 18 | 11.67 (10.34) | 9.67 (7.77) | 13.00 (7.86) | 11.56 (6.13) |
| Education | Bachelor degree | 385 | 9.94 (8.85) | 9.11 (8.07) | 12.28 (8.71) | 9.37 (6.26) |
|  | Postgraduate degree or above | 27 | 9.04 (7.47) | 8.37 (8.27) | 11.33 (10.06) | 8.52 (6.84) |
|  | Junior college | 79 | 7.77 (7.74) | 7.67 (7.31) | 9.90 (8.18) | 8.06 (5.41) |
|  | High school or technical secondary school | 3 | 8.00 (5.29) | 4.67 (2.31) | 10.67 (2.31) | 9.67 (2.89) |
| Professional title | Senior | 41 | 8.83 (7.96) | 6.93 (6.39) | 11.02 (9.13) | 9.56 (6.95) |
|  | Intermediate | 220 | 10.22 (8.71) | 9.23 (8.38) | 12.75 (8.90) | 10.12 (6.49) |
|  | Junior | 193 | 9.01 (8.76) | 8.97 (8.11) | 11.26 (8.64) | 8.30 (5.60) |
|  | No professional title | 40 | 9.05 (7.97) | 7.70 (5.81) | 10.40 (7.24) | 7.10 (5.05) |
| Years of work | >20 | 150 | 9.81 (8.54) | 8.57 (8.04) | 12.11 (9.09) | 10.07 (6.48) |
|  | 3-5 | 48 | 8.96 (9.09) | 8.92 (8.07) | 11.21 (8.83) | 8.54 (6.39) |
|  | 11-20 | 174 | 10.17 (8.82) | 9.46 (8.28) | 12.47 (8.58) | 9.67 (6.32) |
|  | 6-10 | 98 | 9.16 (8.49) | 8.33 (7.56) | 11.35 (8.48) | 7.79 (5.06) |
|  | <=2 | 23 | 6.09 (6.31) | 7.57 (6.52) | 9.13 (7.86) | 5.48 (4.42) |
| Shift pattern | Day shift | 285 | 8.60 (7.56) | 7.89 (7.19) | 11.22 (8.09) | 8.55 (5.99) |
|  | Evening or night shift | 108 | 9.43 (9.44) | 9.56 (8.89) | 11.31 (9.19) | 8.58 (5.70) |
|  | On-call shift | 101 | 12.30 (9.88) | 10.63 (8.62) | 14.14 (9.55) | 11.30 (6.64) |

**Supplementary Table S2B. Observed MBI-GS dimension scores by participant characteristics**

| Characteristic | Category | n | Exhaustion | Cynicism | Professional efficacy |
| --- | --- | --- | --- | --- | --- |
| Hospital type | Tertiary hospital | 258 | 2.44 (1.61) | 2.13 (1.35) | 3.71 (1.24) |
|  | Community health-service centre | 140 | 2.82 (1.63) | 2.57 (1.50) | 3.55 (1.20) |
|  | Secondary hospital | 96 | 2.60 (1.60) | 2.15 (1.38) | 3.62 (1.12) |
| Job category | Nurse | 212 | 2.44 (1.52) | 2.10 (1.31) | 3.63 (1.16) |
|  | Physician | 109 | 3.11 (1.71) | 2.80 (1.57) | 3.56 (1.25) |
|  | Administrative staff | 43 | 2.42 (1.59) | 2.15 (1.22) | 3.70 (1.08) |
|  | Medical technician | 107 | 2.39 (1.62) | 2.12 (1.40) | 3.70 (1.28) |
|  | Logistics staff | 23 | 2.48 (1.66) | 2.08 (1.26) | 3.93 (1.31) |
| Department | Administrative department | 46 | 2.57 (1.64) | 2.29 (1.23) | 3.70 (1.00) |
|  | Surgery | 15 | 2.39 (1.51) | 2.24 (1.51) | 3.73 (1.10) |
|  | Outpatient department | 57 | 2.67 (1.74) | 2.54 (1.42) | 3.68 (1.13) |
|  | Internal medicine | 87 | 2.81 (1.51) | 2.26 (1.38) | 3.40 (1.13) |
|  | Obstetrics and gynaecology | 15 | 2.43 (1.56) | 2.28 (1.20) | 3.57 (1.07) |
|  | Laboratory or examination department | 63 | 2.52 (1.60) | 2.15 (1.27) | 3.92 (1.24) |
|  | Other | 101 | 2.48 (1.65) | 2.28 (1.51) | 3.65 (1.29) |
|  | Operating room | 17 | 2.31 (1.24) | 1.60 (0.71) | 4.05 (1.11) |
|  | Emergency department | 38 | 2.79 (1.91) | 2.30 (1.69) | 3.70 (1.37) |
|  | Logistics department | 14 | 2.24 (1.75) | 1.60 (0.98) | 3.74 (1.38) |
|  | General department | 24 | 2.44 (1.29) | 2.71 (1.71) | 3.32 (1.24) |
|  | Intensive care unit | 17 | 2.46 (1.90) | 2.12 (1.49) | 3.51 (1.36) |
| Sex | Female | 414 | 2.52 (1.57) | 2.21 (1.38) | 3.68 (1.17) |
|  | Male | 80 | 2.88 (1.82) | 2.56 (1.52) | 3.46 (1.38) |
| Age group | 41-50 | 145 | 2.86 (1.70) | 2.53 (1.56) | 3.74 (1.23) |
|  | 20-30 | 109 | 2.48 (1.56) | 2.11 (1.38) | 3.61 (1.17) |
|  | 31-40 | 195 | 2.56 (1.62) | 2.25 (1.32) | 3.50 (1.18) |
|  | >50 | 45 | 2.01 (1.32) | 1.84 (1.18) | 4.10 (1.25) |
| Marital status | Married | 390 | 2.58 (1.61) | 2.28 (1.38) | 3.69 (1.18) |
|  | Unmarried | 86 | 2.52 (1.63) | 2.14 (1.52) | 3.48 (1.28) |
|  | Divorced | 18 | 2.82 (1.68) | 2.52 (1.50) | 3.59 (1.29) |
| Education | Bachelor degree | 385 | 2.67 (1.64) | 2.35 (1.44) | 3.65 (1.20) |
|  | Postgraduate degree or above | 27 | 3.08 (1.42) | 2.21 (1.02) | 3.86 (1.01) |
|  | Junior college | 79 | 1.98 (1.44) | 1.89 (1.30) | 3.59 (1.26) |
|  | High school or technical secondary school | 3 | 2.13 (1.03) | 1.67 (1.53) | 3.17 (2.02) |
| Professional title | Senior | 41 | 2.84 (1.81) | 2.37 (1.41) | 3.89 (1.18) |
|  | Intermediate | 220 | 2.64 (1.67) | 2.36 (1.48) | 3.72 (1.18) |
|  | Junior | 193 | 2.54 (1.53) | 2.18 (1.36) | 3.56 (1.16) |
|  | No professional title | 40 | 2.17 (1.54) | 2.00 (1.23) | 3.41 (1.51) |
| Years of work | >20 | 150 | 2.63 (1.63) | 2.39 (1.48) | 3.89 (1.24) |
|  | 3-5 | 48 | 2.52 (1.52) | 2.19 (1.37) | 3.35 (1.22) |
|  | 11-20 | 174 | 2.59 (1.67) | 2.23 (1.39) | 3.56 (1.18) |
|  | 6-10 | 98 | 2.50 (1.62) | 2.20 (1.39) | 3.50 (1.15) |
|  | <=2 | 23 | 2.71 (1.41) | 2.11 (1.25) | 3.97 (1.11) |
| Shift pattern | Day shift | 285 | 2.42 (1.56) | 2.18 (1.35) | 3.76 (1.23) |
|  | Evening or night shift | 108 | 2.61 (1.66) | 2.11 (1.40) | 3.56 (1.21) |
|  | On-call shift | 101 | 2.99 (1.66) | 2.65 (1.53) | 3.43 (1.11) |
